# *Pseudomonas azotoformans* Belonging to *Pseudomonas fluorescens* Group as Causative Agent of Blue Coloration in Carcasses of Slaughterhouse Rabbits

**DOI:** 10.3390/ani10020256

**Published:** 2020-02-06

**Authors:** Elena Circella, Antonella Schiavone, Roberta Barrasso, Antonio Camarda, Nicola Pugliese, Giancarlo Bozzo

**Affiliations:** Department of Veterinary Medicine, University of Bari - Aldo Moro, Str. Prov. per Casamassima, km 3, 70010 Valenzano (BA), Italy; antonella.schiavone88@hotmail.it (A.S.); roberta.barrasso@uniba.it (R.B.); antonio.camarda@uniba.it (A.C.); nicola.pugliese@uniba.it (N.P.); giancarlo.bozzo@uniba.it (G.B.)

**Keywords:** *Pseudomonas fluorescens* group, *Pseudomonas azotoformans*, carcass contamination, slaughterhouse, rabbits

## Abstract

**Simple Summary:**

Bacteria belonging to the genus *Pseudomonas* are well known for their ubiquitous distribution and their high adaptation capability, which allows them to survive in a wide range of temperatures and other environmental conditions. Therefore, they may colonize food, and a number of cases of food contamination due to *Pseudomonas* spp. have been reported. Among them, in recent years, blue pigmentation due to *Pseudomonas fluorescens* has been widely described in mozzarella cheese, insomuch that it was dubbed the “blue mozzarella” case. Here, we report on the contamination of rabbit meat due to a member of the *P. fluorescens* group that conferred blue coloration to the food matrix. Specifically, colored meat was observed in the refrigeration cell of two butcher shops which had originated from the same slaughterhouse. Bacteriological sampling was performed on pigmented rabbit carcasses as well as from the labeling gun, knives, and water from the slaughterhouse. The same kind of bacterial colony was observed to grow from carcasses, labeling gun, and water. The first identification, performed using a miniaturized biochemical test, revealed it belonged to the *P. fluorescens* group, and further analysis of the 16S ribosomal RNA gene led to definitive identification as *Pseudomonas azotoformans*. These findings highlight the importance of considering the members of the genus *Pseudomonas* and, more specifically, of the *P. fluorescens* group when the microbiological quality of food is to be ascertained.

**Abstract:**

The study describes the finding of an abnormal blue-tinged color found on rabbit carcasses in the refrigeration cell of two butcher shops in Apulia Region. The carcasses were from an industrial rabbitry for production of meat with a regularly authorized slaughterhouse. *Pseudomonas azotoformans*, a microorganism included in *Pseudomonas* *fluorescens* group, was isolated from samples collected by the altered carcasses, showing the growth of uniform bacterial colonies with fluorescent pigmentation. The bacterium was also isolated from an additional water sample and from the labelling gun collected in the slaughterhouse, whilst the knives used for slaughtering resulted negative. Chromatic alteration was experimentally reproduced on new carcasses using a 10^8^ cfu/mL bacterial suspension prepared with the isolated strain. Due to their resistance characteristics, members of *P. fluorescens* group are very difficult to eradicate once introduced into the production environment. Therefore, their presence, even if not considered a public health problem, should be monitored by food industry operators in self-control plans.

## 1. Introduction

The genus *Pseudomonas* includes several rod cell, Gram-negative, aerobic, mesophilic and psychrotolerant bacteria with strictly respiratory metabolism. Their optimum growth temperature is equal to 25 °C, but they can survive in low temperatures [1]. Members of this genus appear as straight or slightly curved bacilli, from 0.5 to 1.0 μm in diameter and 1.5 to 5.0 μm in length, usually mobile for the presence of one or more flagella, and unable to grow at a pH lower than 4.5 [2]. These bacteria are commonly found in decaying organic material like rotting leaves and soil and possess simple nutritional requirements [3,4], constituting up to 90% of the total microbial flora of food.

The genus *Pseudomonas* includes several species, such as *P. aeruginosa*, *P. fluorescens*, and *P. alcaligens*, all which are regarded as human opportunistic pathogens, chiefly in immune-deficient and/or nosocomial patients [5,6], although some species are pathogenic for plants (*P. pseudoalcaligenes*, *P. savastanoi*, *P. syringae*) or for animals (*P. anguilliseptica*, *P. chloraphys*, *P. aeruginosa*) [7].

Although most *Pseudomonas* species have an environmental origin, different species are often observed in foods, depending on the substrate: (i) in milk, *P. ludensis, P. fragi*, *P. fluorescens*, and *P. gessardi* are commonly observed [8]; (ii) in meat, in processing facilities such as cutting and processing laboratories, it is common to observe *P. fluorescens* and *P. fragi* [9]; and (iii) in fish products, *P. aeruginosa*, *P. putida*, *P. chloraphis*, and *P. fluorescens* are reported more frequently, all of which are considered opportunistic for fish species [10,11]. The presence of *P. fluorescens* in food often triggers chromatic alterations, due to enzymatic reactions and production of pigments [12]. Other pigment-producing strains are *P. aeruginosa*, *P. lundensis*, *P. putida*, *P. clororaphis* subsp. *Chlororaphis*, and *P. chlororaphis* subsp. *aureofaciens* [13].

In this study we describe a unique, superficial meat alteration, consisting of blue coloring, observed in rabbit carcasses, and ascribed to a microorganism belonging to *P. fluorescens* group.

## 2. Case History

**Figure 1:**
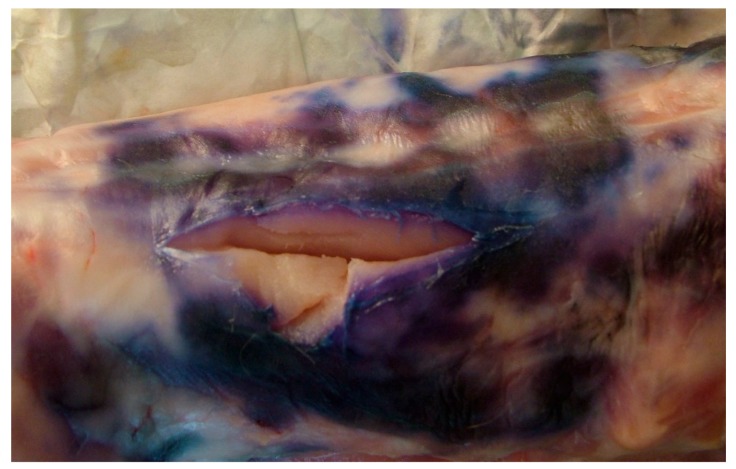
Chromatic alteration observed on the surface of the carcasses.

In the refrigeration cell of two butcher shops in Apulia Region (Southern Italy), rabbit carcasses with a blue meat color were reported (Figure 1). The rabbits were commercial hybrids and came from two different buildings of the same industrial rabbitry for production of meat. They were from the same fattening period, without any pre-slaughter problems. The farm of origin consisted of 1200 holes/brood mares. Out of seven sheds, three were destined for reproduction of breeders and four were used for the fattening stages, with a total production of about 10,000 meat rabbits per month. The slaughterhouse was regularly authorized [14] and was located in the same facilities as the rabbitry. This allowed substantial advantages and economies, as it was not necessary to transport live animals [15]. Additionally, the product was mainly sold and distributed on a local basis, with a maximum commercialization range of 100 km.

Among the 11 butcher shops supplied by the slaughterhouse, in two independent shops chromatic alteration occurred in the cold rooms (at a temperature of 4–6 °C) about 72 h after the arrival of the carcasses. The alteration tended to extend from the inoculation point of the label to form a blue spot 6–7 cm in size and about 2 mm in depth (Figure 1). Anomalies were not observed in the cold rooms of the other sales outlets supplied by the same farm, nor in the cold room of the slaughterhouse. In the butcher shops where the chromatic alteration occurred, the carcasses were stored in the cold rooms (at a temperature of 4 °C ± 2 °C) for about five days before sale, whilst in the other butcher shop, the carcasses were usually stored in the cold room only for 24/36 h before sale.

After observing this alteration, four carcasses were sent to the Avian Pathology Section (Department of Veterinary Medicine, University of Bari, Italy) for laboratory investigation. Additional samples were taken in the slaughterhouse, including: (i) the labelling gun; (ii) the knives used during slaughtering; (iii) the water used for washing the equipment.

## 3. Laboratory Investigations

Both direct and post-enrichment bacteriological tests were performed on the carcasses. Sterile swabs, humidified in sterile physiological solution, were rubbed over the blue spots of the carcasses for direct examination. The swabs were passed onto Trypticase Soy Agar (TSA-Oxoid, Milan, Italy) enriched media and selective media (Pseudomonas Agar Base-Oxoid, Milan, Italy). At the same time, portions of tissues were placed in pre-enrichment in peptone water (ratio of 1:10). After 24 h of incubation at 37 °C, the broths were seeded onto solid media, TSA, and Pseudomonas Agar Base. Sterile swabs, humidified in sterile physiological solution, were rubbed over the knives and labelling gun and seeded onto TSA and Pseudomonas Agar Base. For the analysis of the slaughterhouse water, 100 µL of water was seeded onto TSA and Pseudomonas Agar Base. Liquid and solid media were incubated under aerobic conditions at 37 °C. The incubation time was 24 h for each step. Isolation of the colonies was performed on TSA and their identification was obtained by biochemical tests in micro-method (Api 20NE tunnels-BioMerieux).

In order to confirm the identification, a colony-PCR targeting the 16S rRNA gene was carried out. Briefly, a single, well-isolated colony from a pure culture was picked and resuspended in 10 μL of sterile distilled water. Two microliters of cell suspension were used as a template in the reaction, performed by using the Platinum II Got-Start Green PCR Mastermix (ThermoFisher Scientific, Milan, Italy) and adding 0.75 μM each of 27F (5′-AGAGTTTGATCMTGGCTCAG-3′) and 1492R (5′-CTACGRVTACCTTGTTACGAC-3′) primers (modified from [16]). The gathered amplicon was purified by the mean of the PureLink Quick Gel Extraction and PCR Purification Combo Kit (ThermoFisher Scientific) and sequenced by the BigDye Terminator method at the facilities of Bio-Fab Research (Rome, Italy). Other than PCR primers, the 341f (5′-CCTACGGGAGGCAGCAG-3′) and 907r (5′-CCCCGTCAATTCATTTGAGTTT-3′) primers [17] were used for sequencing. The reads were assembled by the online Cap3 Sequence Assembly Program [18] and the final nucleotide sequence, after removal of primers and low quality regions, was submitted to GenBank under the accession number MN807243.

The sequence was compared by BLAST with those available in GenBank from type materials.

The sequence was aligned with a panel of 102 representative 16S rRNA gene nucleotide sequences from *Pseudomonas* spp., especially selecting reference sequences belonging to the *P. fluorescens* complex, sequences from type strains, and from strains described in food contamination reports. The complete list of sequences is provided in Supplementary Table S1. The sequence from *Cellvibrio japonicus* was also included as an outgroup. Alignment was performed by the ClustalW algorithm implemented in MEGA v10.1.6 [19]. Phylogeny was inferred by PhyML 3.0 [20] (with bootstrap calculated from 1000 replicates) according to the General Time Reversible plus Gamma (K = 4, α = 0.12213) evolutionary model, selected by using the web implementation of Model Test [21], available at the URL https://www.hiv.lanl.gov/content/sequence/findmodel/findmodel.html.

Using the isolated bacterial colonies, experimental reproduction of the chromatic alteration was performed preparing a bacterial suspension with 10^8^ cfu/mL, according to the bacterial load (>10^7^ cfu/g) generally found in other matrixes with blue alteration due to *P. fluorescens* [22]. The suspension was spread by brushing with sterile swabs onto the surface of two rabbit carcasses. The carcasses were immediately placed in the cold room at a temperature of about 4 °C, and were kept under observation for the following three days. Moreover, the microorganism was re-isolated and identified out of the experimentally infected meat in order to confirm the agent of the observed macroscopic lesions.

## 4. Results

The bacteriological tests, performed on the samples collected by the altered carcasses, showed the growth of uniform bacterial colonies with the fluorescent pigmentation typical of *P. fluorescens* on the enriched soil. A microorganism belonging to *P. fluorescens* group was identified in the examined meat samples. The bacterium was also isolated from the water sample and from the labelling gun, whilst the knives used for slaughtering resulted negative.

The BLAST analysis of the nucleotide sequence of the 16S rRNA gene revealed it was 99.93% identical to the corresponding sequence of the strain IAM 1603 of *Pseudomonas azotoformans* (accession numbers LC130639 and LC130640), and 99.86% identical to those from *Pseudomonas azotoformans* strain LGM 21611 (accession number LT629702) and from *Pseudomonas paralactis* strain DSM 29164 (RefSeq accession number NR_156987.1).

**Figure 2:**
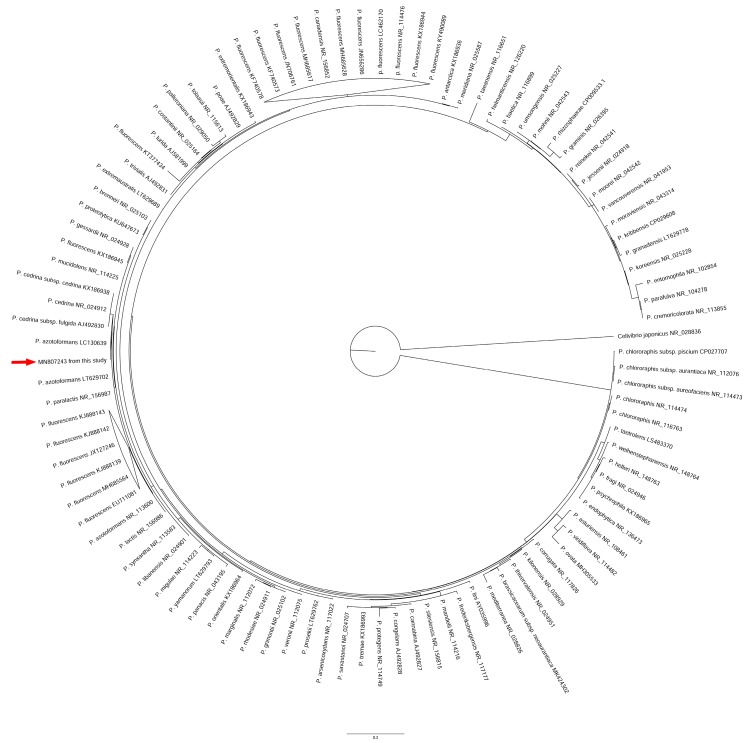
Maximum likelihood phylogenetic tree based on the nucleotide sequence of the 16S rRNA genes among members of the *Pseudomonas* genus. Red arrow indicates the sequence from this study.

The phylogenetic analysis confirmed the close evolutionary relationship between the isolate from this study and *P. azotoformans* IAM 1603, as they constituted a well-separated subgroup with respect to *Pseudomonas cedrina*, and were distinct from the subgroup including *P. paralactis*, *Pseudomonas lactis,* and other strains of *P. azotoformans* (Figure 2 and Figure S1).

**Figure 3:**
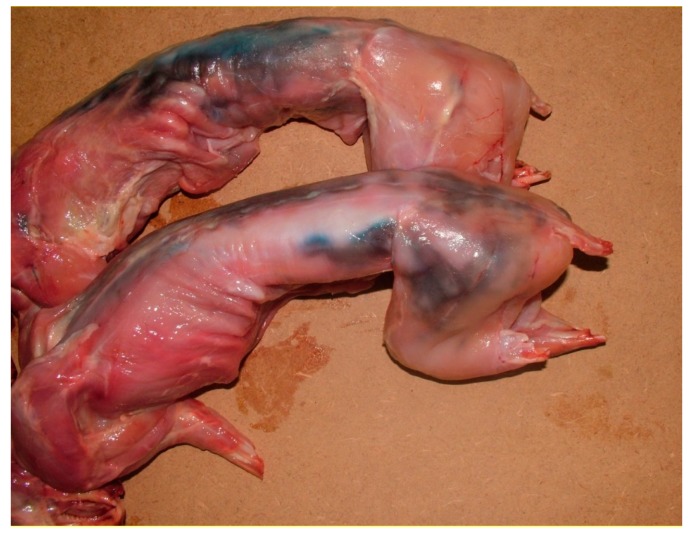
Chromatic alteration experimentally reproduced.

Experimental reproduction of the chromatic alteration was obtained as soon as 24 h after the superficial contamination (Figure 3). The alteration was similar to that observed in the cold rooms of the butcher shops under investigation. Therefore, the chromatic alteration was related to the presence of a microorganism very close to *P. azotoformans*.

## 5. Discussion

The isolate from this study, causing the blue coloration of the rabbit meat, was found to be strictly related to *P. azotoformans*, a species, in turn, very close to the denitrifying biovars of *P. fluorescens* [23], insomuch that it has been included in the *P. fluorescens* lineage of the *P. fluorescens* group [24], and, more recently, located within the *P. fluorescens* phylogenomic group [16].

As outlined above, *P. fluorescens* has been frequently found in food substrates, and it is well known for having been the cause of spoilage of dairy products, such as mozzarella, which exhibited a very typical blue coloration [25,26]. Unfortunately, to our knowledge, no 16S rRNA gene sequences from those strains are available in GenBank for comparison, so it has not been possible to highlight possible similarities or differences with the strains causing those events.

However, the isolation of *P. azotoformans* from rabbit meat remains noteworthy, since that species has not yet been found to be associated with food contamination, apart from a recent report of milk contamination [27]. This may help to drive attention to an organism that is relatively unknown, and probably undervalued in its spoilage potential.

As well as the other members of *P. fluorescens* group, itis a microorganism with poor nutritional requirements. Indeed, *P. fluorescens* has the ability to adapt even to hostile environments, e.g., cold rooms, where the conditions for its growth are not optimal [3,4]. The ability of these microorganisms to adapt to different environments is likely accounted for by the formation of biofilms, making the bacterial population very resistant [28,29].

In agreement with other studies [30], the isolation of *P. fluorescens*-related microorganisms from the water sample and from the labelling gun suggests the importance of good sanitization procedures. The abnormal coloration observed on the carcasses started from the label inoculation point, indicating that the carcass contamination was probably due to the labelling gun. Indeed, the primary source of contamination was likely the labelling gun, which was contaminated. Moreover, any meat fragments on the labelling gun could allow for additional replication of pseudomonas or other opportunistic microorganisms after contamination. Accordingly, the labelling gun should be disinfected frequently during slaughtering operations, to improve gun cleaning and decontamination when labelling the carcasses. As previously suggested [30], the alteration induced by *P. fluorescens* was directly correlated to storage time in addition to environmental temperature. In the two butcher shops under investigation, there was a longer storage time in the cold rooms (of about five days) before sale than in the other butcher shops, where the carcasses were stored for less than 24/36 h. Moreover, bacterial load seems to play a role in the appearance time of the alteration. In fact, the chromatic alteration appeared as soon as 24 h after contamination with a suspension of 10^8^ cfu/mL under the above-mentioned experimental conditions.

Although *P. fluorescens* and *P. azotoformans* are rarely, if never, associated with human pathologies, there are several reports in which the presence of *P. fluorescens* in dairy products, fish, vegetables, and meat has led to marked deterioration of the products and withdrawal from the market [26,31,32].

Due to its resistance characteristics, *P. fluorescens* is very difficult to eradicate once introduced into the production environment [1]. Therefore, although it is not considered therein, it could be regarded as a “process hygiene criterion” under Commission Regulation (EC) No. 2073/2005 [33] as an environmental contaminant, just like *Enterobacteriaceae*. Consequently, although not mentioned in food regulations, contamination due to species belonging to *P. fluorescens* group should be considered unacceptable because it makes food unsuitable for human consumption. Therefore, any products altered by those organisms should be withdrawn from the market in agreement with Regulation (EC) No. 178/2002 of the European Parliament and of the Council [34]. Accordingly, even though the presence of *Pseudomonas* spp. is not considered a public health problem, it should in any case be monitored by food industry operators in their self-control plans.

## 6. Conclusions

Considering its high adaptability and its resistance characteristics, *P. fluorescens* is a very difficult microorganism to eradicate once introduced into the production environment. Also taking into account the possibility of *P. fluorescens* contamination of food matrixes of animal origin, it should be advisable that it could be regarded as a “process hygiene criterion” under Commission Regulation (EC) No 2073/2005 [33] as an environmental contaminant, just like Enterobacteriaceae. Consequently, although not mentioned in food regulations, contamination due to species belong to *P. fluorescens* should be considered unacceptable because it makes food unsuitable for human consumption. Therefore, any products altered by those organisms should be withdrawn from the market in agreement with Regulation (EC) No 178/2002 of the European Parliament and of the Council [34]. Accordingly, even though the presence of *Pseudomonas* spp. is not considered a public health problem, it should, in any case, be monitored by food industry operators in their self-control plans.

## Supplementary Materials

The following are available online at https://www.mdpi.com/2076-2615/10/2/256/s1, Figure S1: Cladrogram showing the phylogenetic relationships among the 16S rRNA sequences from microorganisms belonging to the genus *Pseudomonas*. The sequence from this study is highlighted by a red arrow. Bootstrap values are reported at nodes, Table S1: List of 16S rRNA gene sequences used in this study.
