# Supplementary material for: Pseudomonas azotoformans Belonging to Pseudomonas fluorescens Group as Causative Agent of Blue Coloration in Carcasses of Slaughterhouse Rabbits
Source: Animals (Basel). 2020 Feb 6;10(2):256. doi: 10.3390/ani10020256 (PMC7070765; doi:10.3390/ani10020256)
Supplement: Supplementary file 1 [file animals-10-00256-s001.zip › Table S1.docx]

**Supplementary Table S1.** List of 16S rRNA gene sequences used in this study.

| ***GenBank accession number*** | ***Species*** | ***Strain*** | ***Source*** |
| --- | --- | --- | --- |
| AJ492827.1 | *Pseudomonas cannabina* | CFBP 2341T | Phyllosphere |
| AJ492828.1 | *Pseudomonas congelans* | DSM 14939T | Phyllosphere |
| AJ492829.1 | *Pseudomonas poae* | DSM 14936T | Phyllosphere |
| AJ492830.1 | *Pseudomonas cedrina*  *subsp. fulgida* | DSM 14938T | Phyllosphere |
| AJ492831.1 | *Pseudomonas trivialis* | DSM 14937T | Phyllosphere |
| AJ581999.1 | *Pseudomonas lurida* | DSM 15835 | Phyllosphere |
| AY035996.2 | *Pseudomonas lini* | CFBP 5737 | Soil |
| CP009533.1 | *Pseudomonas rhizosphaerae* | DSM 16299 | Soil |
| CP027707.1 | *Pseudomonas chlororaphis*  *subsp. piscium* | DSM 21509 | Fish |
| CP029608.1 | *Pseudomonas kribbensis* | 46-2 | Soil |
| EU711081.1 | *Pseudomonas fluorescens* | EBL-1 | Fungi |
| JN656286.1 | *Pseudomonas fluorescens* | Is7 | Arthropod |
| JN706761.1 | *Pseudomonas fluorescens* | Cb3 | Arthropod |
| JX127246.1 | *Pseudomonas fluorescens* | 4.9.3 | Marine sediment |
| KF740573.1 | *Pseudomonas fluorescens* | Xg4 | Arthropod |
| KF740578.1 | *Pseudomonas fluorescens* | Xg9 | Arthropod |
| KJ888139.1 | *Pseudomonas fluorescens* | Bp-11 | Arthropod |
| KJ888142.1 | *Pseudomonas fluorescens* | Bp-14 | Arthopod |
| KJ888143.1 | *Pseudomonas fluorescens* | Bp-15 | Arthopod |
| KT377434.1 | *Pseudomonas fluorescens* | Dk3 | Arthropod |
| KU647673.1 | *Pseudomonas proteolytica* | BD13-00014 | Water |
| KX186936.1 | *Pseudomonas antarctica* | DSM:15318 | Type strain |
| KX186938.1 | *Pseudomonas cedrina*  *subsp. cedrina* | DSM:17516 | Type strain |
| KX186943.1 | *Pseudomonas extremorientalis* | DSM:15824 | Type strain |
| KX186944.1 | *Pseudomonas fluorescens* | DSM 50090 | Type strain |
| KX186945.1 | *Pseudomonas fluorescens* | DSM 50124 | Type strain |
| KX186964.1 | *Pseudomonas orientalis* | DSM:17489 | Type strain |
| KX186965.1 | *Pseudomonas psychrophila* | DSM:17535 | Type strain |
| KX186993.1 | *Pseudomonas tremae* | DSM:16744 | Type strain |
| KY490089.1 | *Pseudomonas fluorescens* | TBS09 | Marine sediment |
| LC130639.1 | *Pseudomonas azotoformans* | IAM 1603 | Type strain |
| LC462170.1 | *Pseudomonas fluorescens* | JCM 5963 | Type strain |
| LS483370.1 | *Pseudomonas taetrolens* | NCTC10697 | Type strain |
| LS483433.1 | *Pseudomonas mucidolens* | NCTC8068 | Type strain |
| LT629689.1 | *Pseudomonas extremaustralis* | DSM 17835 | Type strain |
| LT629702.1 | *Pseudomonas azotoformans* | LMG 21611 | Type strain |
| LT629762.1 | *Pseudomonas prosekii* | LMG 26867 | Type strain |
| LT629778.1 | *Pseudomonas granadensis* | LMG 27940 | Type strain |
| LT629793.1 | *Pseudomonas yamanorum* | LMG 27247 | Type strain |
| MH305533.1 | *Pseudomonas ovata* | F51 | Skin ulcer |
| MH685564.1 | *Pseudomonas fluorescens* | 6.1 | Soil |
| MH685617.1 | *Pseudomonas fluorescens* | 11.1 | Soil |
| MH685628.1 | *Pseudomonas fluorescens* | 15.1 | Soil |
| MK424302.1 | *Pseudomonas brassicacearum*  *subsp. neoaurantiaca* | ATCC 49054 | Soil |
| NR_024707.1 | *Pseudomonas savastanoi* | ATCC 13522 | Type strain |
| NR_024901.1 | *Pseudomonas libanensis* | CIP 105460 | Water |
| NR_024911.1 | *Pseudomonas rhodesiae* | CIP 104664 | Water |
| NR_024912.1 | *Pseudomonas cedrina* | CFML 96-198 | Water |
| NR_024918.1 | *Pseudomonas jessenii* | CIP 105274 | Water |
| NR_024928.1 | *Pseudomonas gessardii* | CIP 105469 | Water |
| NR_024946.1 | *Pseudomonas fragi* | ATCC 4973 | Type strain |
| NR_024951.1 | *Pseudomonas thivervalensis* | SBK26 | Soil |
| NR_025102.1 | *Pseudomonas grimontii* | CFML 97-514 | Type strain |
| NR_025103.1 | *Pseudomonas brenneri* | CFML 97-391 | Water |
| NR_025164.1 | *Pseudomonas costantinii* | CFBP 5705 | Fungi |
| NR_025227.1 | *Pseudomonas umsongensis* | Ps 3-10 | Soil |
| NR_025228.1 | *Pseudomonas koreensis* | Ps 9-14 | Type strain |
| NR_025587.1 | *Pseudomonas meridiana* | CMS 38 | Type strain |
| NR_026395.1 | *Pseudomonas graminis* | DSM 11363 | Plant |
| NR_028826.1 | *Pseudomonas mediterranea* | CFBP 5447 | Type strain |
| NR_028836.1 | *Cellvibrio japonicus* | Ueda107 | Type strain |
| NR_028929.1 | *Pseudomonas kilonensis* | 520-20 | Soil |
| NR_029050.1 | *Pseudomonas palleroniana* | CFBP 4389 | Soil |
| NR_041953.1 | *Pseudomonas vancouverensis* | DhA-51 | Type strain |
| NR_042541.1 | *Pseudomonas reinekei* | MT1 | Type strain |
| NR_042542.1 | *Pseudomonas moorei* | RW10 | Type strain |
| NR_042543.1 | *Pseudomonas mohnii* | IpA-2 | Type strain |
| NR_043195.1 | *Pseudomonas panacis* | CG20106 | Plant |
| NR_043314.1 | *Pseudomonas moraviensis* | 1B4 | Soil |
| NR_102854.1 | *Pseudomonas entomophila* | L48 | Soil |
| NR_104278.1 | *Pseudomonas parafulva* | DSM 17004 | Type strain |
| NR_108461.1 | *Pseudomonas asturiensis* | LPPA 221 | Plant |
| NR_112072.1 | *Pseudomonas marginalis* | ATCC 10844 | Type strain |
| NR_112075.1 | *Pseudomonas veronii* | CIP 104663 | Type strain |
| NR_112076.1 | *Pseudomonas chlororaphis*  *subsp. aurantiaca* | ATCC 33663 | Type strain |
| NR_113583.1 | *Pseudomonas synxantha* | NBRC 3913 | Type strain |
| NR_113600.1 | *Pseudomonas azotoformans* | NBRC 12693 | Type strain |
| NR_113855.1 | *Pseudomonas cremoricolorata* | NBRC 16634 | Type strain |
| NR_114216.1 | *Pseudomonas mandelii* | NBRC 103147 | Type strain |
| NR_114223.1 | *Pseudomonas migulae* | NBRC 103157 | Type strain |
| NR_114225.1 | *Pseudomonas mucidolens* | NBRC 103159 | Type strain |
| NR_114473.1 | *Pseudomonas chlororaphis*  *subsp. aureofaciens* | ATCC 13985 | Type strain |
| NR_114474.1 | *Pseudomonas chlororaphis* | ATCC 9446 | Type strain |
| NR_114476.1 | *Pseudomonas fluorescens* | ATCC 13525 | Type strain |
| NR_114482.1 | *Pseudomonas viridiflava* | ATCC 13223 | Type strain |
| NR_114749.1 | *Pseudomonas protegens* | CHA0 | Plant |
| NR_115613.1 | *Pseudomonas tolaasii* | ATCC 33618 | Type strain |
| NR_116651.1 | *Pseudomonas taeanensis* | MS-3 | Environment |
| NR_116763.1 | *Pseudomonas chlororaphis* | ATCC 9446 | Type strain |
| NR_116899.1 | *Pseudomonas baetica* | a390 | Soleidae |
| NR_117022.1 | *Pseudomonas arsenicoxydans* | VC-1 | Environment |
| NR_117177.1 | *Pseudomonas frederiksbergensis* | DSM 13022 | Type strain |
| NR_117826.1 | *Pseudomonas corrugata* | CFBP:2431 | Plant |
| NR_126220.1 | *Pseudomonas helmanticensis* | OHA11 | Soil |
| NR_136473.1 | *Pseudomonas endophytica* | BSTT44 | Plant |
| NR_148763.1 | *Pseudomonas helleri* | DSM 29165 | Milk |
| NR_148764.1 | *Pseudomonas weihenstephanensis* | DSM 29166 | Milk |
| NR_156815.1 | *Pseudomonas silesiensis* | A3 | Sewage |
| NR_156852.1 | *Pseudomonas canadensis* | 2-92 | Soil |
| NR_156986.1 | *Pseudomonas lactis* | DSM 29167 | Milk |
| NR_156987.1 | *Pseudomonas paralactis* | DSM 29164 | Milk |
