# Supplementary figures and images for: Pseudomonas azotoformans Belonging to Pseudomonas fluorescens Group as Causative Agent of Blue Coloration in Carcasses of Slaughterhouse Rabbits
Source: Animals (Basel). 2020 Feb 6;10(2):256. doi: 10.3390/ani10020256 (PMC7070765; doi:10.3390/ani10020256)

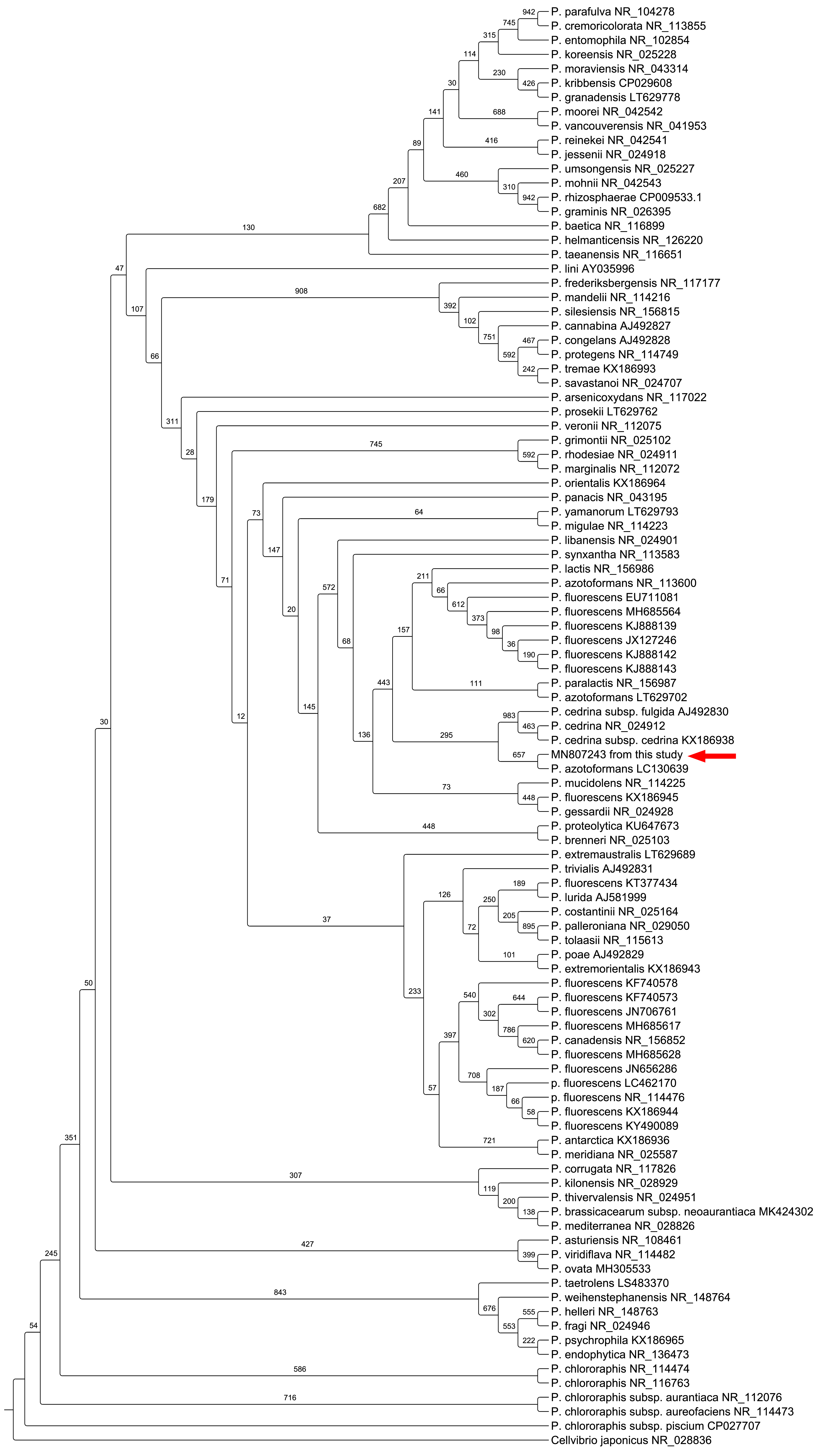

Supplement: Supplementary file 1 [file animals-10-00256-s001.zip › Fig.S1.tif]
